# *OCT3* promoter haplotype is associated with metformin pharmacokinetics in Koreans

**DOI:** 10.1038/s41598-018-35322-6

**Published:** 2018-11-16

**Authors:** Eun Young Kwon, Jae-Yong Chung, Hyo Jin Park, Bo Min Kim, Minsuk Kim, Ji Ha Choi

**Affiliations:** 1 Department of Pharmacology, Tissue Injury Defense Research Center, College of Medicine, Ewha Womans University, Seoul, Korea; 2 Department of Clinical Pharmacology and Therapeutics, Seoul National University College of Medicine and Bundang Hospital, Seoul, Korea

## Abstract

Organic cation transporter 3 (OCT3) is expressed in various organs in humans and plays an important role in the transport of organic cations and drugs including metformin. In this study, we identified genetic variations of the *OCT3* promoter and functionally characterized each variant by *in vitro* assays. Next, the association between the functional haplotype of the *OCT3* promoter and pharmacokinetics of metformin was evaluated. In our study population, 7 variations and 2 major haplotypes were identified, of which H2 haplotype yielded a significantly higher luciferase activity than did the wild type. Two variants of H2, c.-1603G > A and c.-1547T > G, yielded significantly lower luciferase activities, whereas the luciferase activity of another variant, c.-29G > A, was significantly higher. Two transcription factors, Sp1 and USF1, were involved in the regulation of *OCT3* transcription. Analysis of clinical data revealed that 25 subjects, either homozygous or heterozygous for H2, showed increased AUC_inf_ and C_max_ by 17.2% and 15.9%, respectively [*P* = 0.016 and 0.031, GMR (90% CI) = 1.17 (1.06–1.29) and 1.17 (1.04–1.31), respectively], compared to the 20 subjects in the control group. Our study suggests that an *OCT3* promoter haplotype affects the pharmacokinetics of metformin in Koreans as well as the *OCT3* transcription rate.

## Introduction

Organic cation transporter 3 (OCT3), encoded by the solute carrier 22A3 (*SLC22A3*), is expressed in various organs in humans, including skeletal and smooth muscle, liver, heart, intestine, brain, placenta, salivary glands, and kidneys^1–4^. It mediates the transport of 1-methyl-4-phenylpyridium (MPP^+^) and several cationic drugs including metformin^5,6^. OCT3 is also defined as an extraneuronal monoamine transporter because it is known to play an important role in transporting norepinephrine, epinephrine, and histamine^5,7–9^.

To date, few studies have been conducted to identify and functionally characterize the genetic variations of *OCT3*. For example, Chen *et al*.^10^ revealed that a variant (rs555754) of the *OCT3* proximal promoter region yields increased luciferase activity and results in increased expression of OCT3 in the liver. They also reported that expression of OCT3 is low in prostate cancer, and one of the important mechanisms underlying its reduced expression is hypermethylation of the *OCT3* promoter.

Metformin is the most commonly prescribed drug for the treatment of type 2 diabetes mellitus. Interindividual variability of the pharmacokinetics or pharmacodynamics of metformin is large, and various transporters such as OCT1 and −2, plasma membrane monoamine transporter, multidrug and toxin extrusion 1 (MATE1), and MATE2K are involved in this variability^11–20^. For instance, several variants of *OCT1* and *OCT2* have been shown to have significant effects on the pharmacokinetics of metformin^17,18,20,21^. In addition, variants c.-66T > C and c.-130G > A of the *MATE1* and *MATE2K* promoter, respectively, significantly affect renal secretion of metformin, and the c.-130G > A variant is associated with a poorer response to this drug^11,13^. In contrast to transporters OCT1 and −2 or MATE1 and −2K, few studies have been conducted to evaluate the association between *OCT3* variants and the pharmacokinetics or pharmacodynamics of metformin. Tzvetkov *et al*.^20^ investigated the effect of 6 variants of *OCT3*, including 5 variants in introns and 1 synonymous variant, on the renal clearance of metformin, and found none of these variants to be associated with metformin excretion. In addition, 2 studies were conducted to determine an association between *OCT3* variants and response to metformin, and no significant association was found^22,23^. Recently, Shirasaka *et al*.^24^ observed that pharmacokinetic parameters and bioavailability differ significantly between wild type (Oct3^+/+^) and *Oct3* knockout (Oct3^−/−^) mice, and their results imply that Oct3 is involved in the intestinal absorption of metformin. In another study, 3 nonsynonymous variants of *OCT3* were found to significantly alter metformin uptake and kinetics in *in vitro* analyses^1^.

In the present study, we identified genetic variations of the *OCT3* promoter by sequencing DNA samples from 48 healthy Koreans and investigated the effects of common haplotypes by *in vitro* assays. Subsequently, the effect of an *OCT3* promoter haplotype on pharmacokinetics of metformin in 45 healthy Korean volunteers was evaluated.

## Results

### Identification of genetic variations in the *OCT3* promoter

By sequencing the *OCT3* promoter region, we identified 7 promoter variations in our study population (Table 1). Among them, 4 variations, c.-993C > G, c.-423C > A, c.-386C > T, and c.-258C > T, are reported for the first time by this study. In addition, we observed that the frequencies of the 3 known variations, c.-1603G > A, c.-1547T > G, and c.-29G > A were similar to those of Han Chinese in Beijing, China (CHB) (Supplementary Table S1). For the purpose of comparison, the genotype data of *OCT3* variations in other ethnic groups were obtained from the 1000 Genomes Project (phase 3) (https://www.ncbi.nlm.nih.gov/variation/tools/1000genomes/). Table 2 shows the frequency distributions of the *OCT3* haplotypes in Koreans. Among 8 haplotypes, 2 (H1 and H2) showed high frequencies (69.7% and 24.0%, respectively). Using linkage disequilibrium analysis of the single nucleotide polymorphisms (SNPs), we found that c.-1603G > A was in linkage disequilibrium with c.-1547T > G (*r*^2^ = 1.0), and both SNPs were in linkage disequilibrium with c.-29G > A (*r*^2^ > 0.8) (Supplementary Fig. S1). In this study, the H1 haplotype was assumed to be a wild type haplotype according to entry NM_021977.3 in the database of SNPs at the National Center for Biotechnology Information.

**Table 1:** Frequencies of *OCT3* genetic variations in promoter region.

rs Number	Variation	Minor allele	Minor allele frequency	rs Number	Variation	Minor allele	Minor allele frequency
rs520685	c.-1603G > A	A	0.260	—	c.-386C > T	T	0.011
rs520829	c.-1547T > G	G	0.260	—	c.-258C > T	T	0.011
—	c.-993C > G	G	0.021	rs555754	c.-29G > A	A	0.293
—	c.-423C > A	A	0.010				

Data were obtained from DNA samples from 48 unrelated Korean individuals.

The position of the variation is based upon the translational start site.

**Table 2:** Frequencies of *OCT3* promoter haplotypes.

ID	c.-1603 G > A	c.-1547 T > G	c.-993 C > G	c.-423 C > A	c.-386 C > T	c.-258 C > T	c.-29 G > A	Frequency
H1	G	T	C	C	C	C	G	0.697
H2	**A**	**G**	C	C	C	C	**A**	0.240
H3	G	T	C	C	C	C	**A**	0.011
H4	G	T	C	C	**T**	C	G	0.011
H5	G	T	**G**	C	C	C	G	0.011
H6	**A**	**G**	C	**A**	C	C	**A**	0.011
H7	**A**	**G**	C	C	C	**T**	**A**	0.011
H8	G	T	**G**	C	C	C	**A**	0.011

The minor alleles were marked in bold-faced letters with underlines.

### Effects of variants on *OCT3* promoter activity

**Figure 1:**
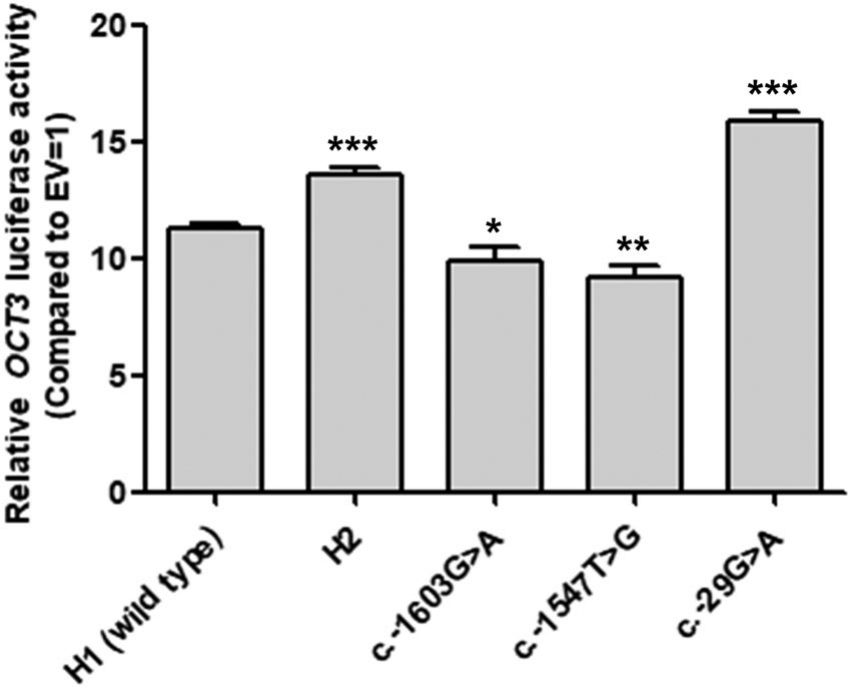
Luciferase activities of the *OCT3* promoter haplotypes or variants. Luciferase activities were measured 48 hours after the transfection of reporter plasmids containing the wild type *OCT3* or its variants into HCT-116 cells. The relative luciferase activity of each vector was compared to that of the empty vector (EV = 1). The data shown represent the mean ± SD from three wells. ^*^P < 0.05, ^**^P < 0.01, ^***^P < 0.001 as compared to EV

To examine the effects of the variants on the promoter activity of *OCT3*, we first constructed reporter plasmids containing major haplotypes H1 or H2 and performed reporter assays. As a result, we observed that the luciferase activity of H2 was 20.6% higher than that of the wild type (H1) (*P* < 0.001, Fig. 1). Then, we measured the luciferase activity of the 3 variants in H2 and found that 2 variants, c.-1603G > A (rs520685) and c.-1547T > G (rs520829), showed significantly decreased luciferase activity by 12.9% and 19.1%, respectively (*P* < 0.05 and 0.01, respectively). On the other hand, the luciferase activity of the other variant, c.-29G > A (rs555754), was significantly increased by 40.6% in agreement with the results of another study^10^ (*P* < 0.001, Fig. 1).

### Transcription factors that regulate the *OCT3* promoter transcriptional activity

**Figure 2:**
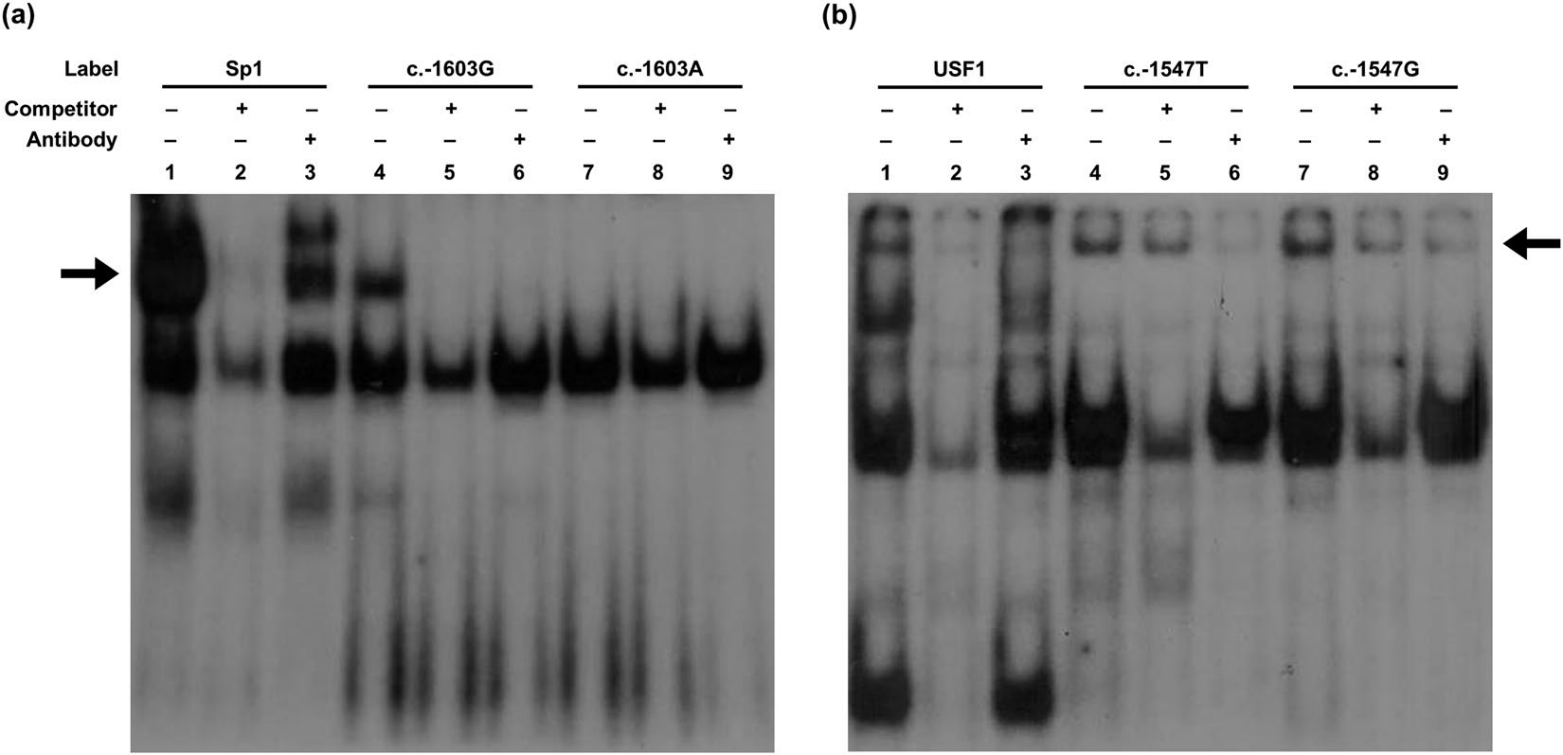
EMSAs for identification of the interaction between *OCT3* promoter variants and transcription factors. (**a**) Nuclear extracts were incubated with ^32^P-labeled oligonucleotides (Sp1 consensus, lanes 1–3; c.-1603G wild type, lanes 4–6; c.-1603A variant, lanes 7–9). Competition assays and supershift assays were conducted with 100-fold molar excess of Sp1 consensus oligonucleotides (lanes 2, 5, and 8) and Sp1 antibody (lanes 3, 6, and 9), respectively. (**b**) Nuclear extracts were incubated with ^32^P-labeled oligonucleotides (USF1 consensus, lanes 1–3; c.-1547T wild type, lanes 4–6; c.-1547G variant, lanes 7–9]. Competition and supershift assays were conducted with 100-fold molar excess of USF1 consensus oligonucleotides (lanes 2, 5, and 8) and USF1 antibody (lanes 3, 6, and 9), respectively. The arrows indicate the position of the DNA–protein complexes. Cropped gels are used. Full-length gels are shown in Supplementary Fig. S3.

**Figure 3:**
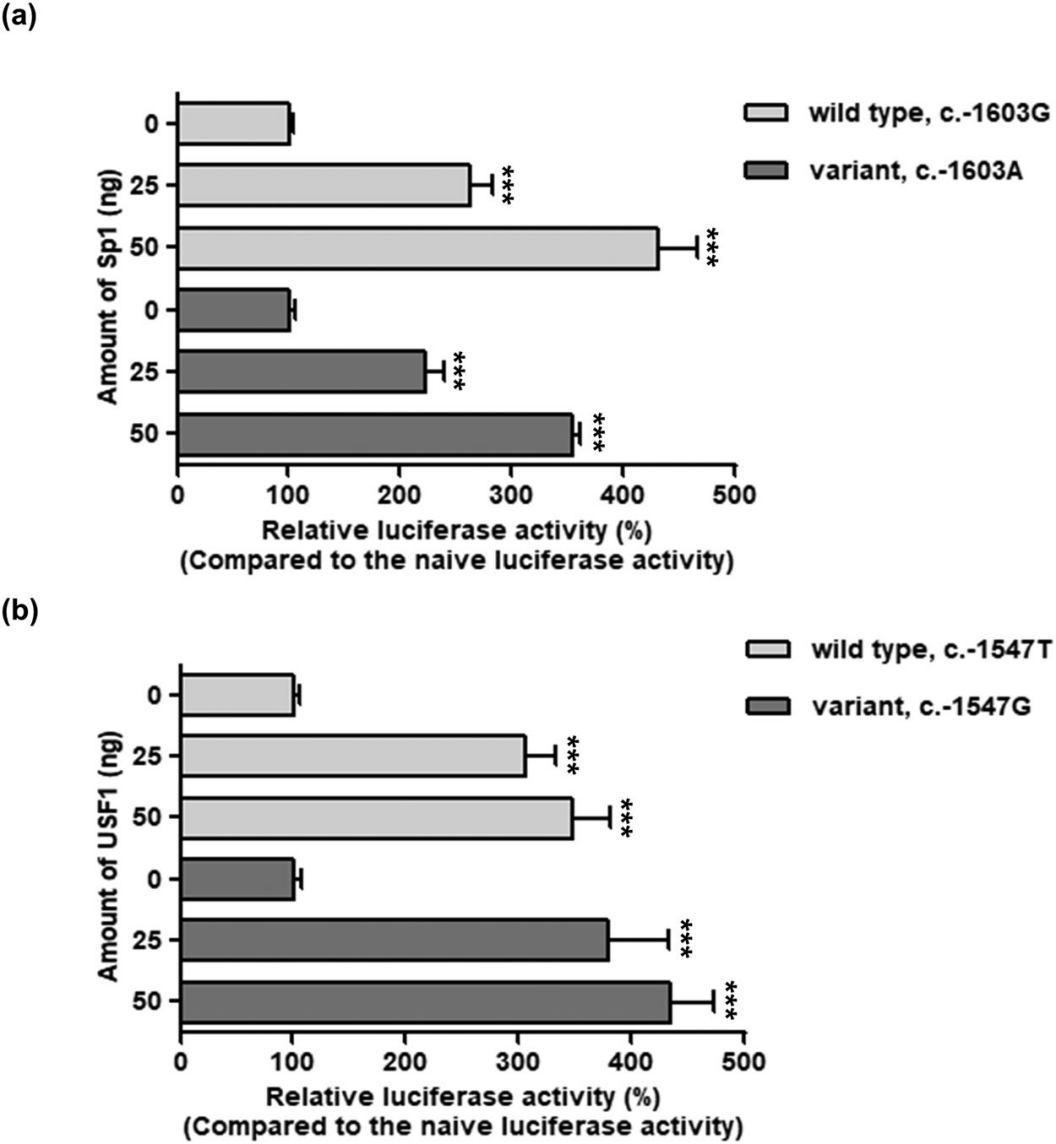
Effect of Sp1 or USF1 on the promoter activity of *OCT3*. Forty-eight hours after co-transfection of wild type *OCT3* or its variant reporter plasmids and various amounts of *Sp1* or *USF1* cDNA into HCT-116 cells, luciferase activities were measured. The luciferase activity of each construct was compared with naïve promoter activity. The data shown represent mean ± SD from three wells. ^***^P < 0.001 as compared to naïve promoter activity.

Chen *et al*.^10^ reported that c.-29G > A interacts with a transcription factor, specificity protein 1 (Sp1). Here, to examine other transcription factors that are responsible for the decreased promoter activity of *OCT3*, we predicted transcription factors that can bind to the *OCT3* promoter near c.-1603G > A or c.-1547T > G using Consite (http://consite.genereg.net) and JASPAR (http://jaspar.genereg.net). As a result, 2 transcription factors, Sp1 and upstream stimulating factor 1 (USF1), were predicted to bind to the promoter region near c.-1603G > A and c.-1547T > G, respectively, and the binding affinity of each transcription factor differed between the wild type and variant sequences. We validated this prediction by electrophoretic mobility shift assays (EMSAs). First, we confirmed the position of the DNA–Sp1 complex in competition or supershift assays after incubation of nuclear proteins with ^32^P-labeled Sp1 consensus oligonucleotides (lanes 1–3, Fig. 2a). Then, we found that Sp1 bound to the c.-1603G wild type sequence much more strongly than to variant c.-1603A (lanes 4 and 7, Fig. 2a). Competition (lanes 5 and 8, Fig. 2a) or supershift (lanes 6 and 9, Fig. 2a) assays confirmed that Sp1 was present in the DNA-protein complex. As for the c.-1547T > G variant, similar to the Sp1 EMSA assay, we confirmed the position of the DNA-USF1 complex in competition or supershift assays after incubation of nuclear proteins with ^32^P-labeled USF1 consensus oligonucleotides (lanes 1–3, Fig. 2b). By incubating nuclear proteins with ^32^P-labeled c.-1547T or c.-1547G oligonucleotides, we observed that USF1 bound to the promoter region near this variant; in particular, the binding affinity was 16.9% stronger in the presence of the c.-1547G variant sequence compared to the c.-1547T wild type sequence (lanes 4 and 7, Fig. 2b). In addition, we confirmed that USF1 was present in the c.-1547T or c.-1547G oligonucleotide-protein complex by competition (lanes 5 and 8, Fig. 2b) or supershift (lanes 6 and 9, Fig. 2b) assays. Finally, we examined the effect of Sp1 or USF1 on the promoter activity of *OCT3* by measuring the luciferase activities of *OCT3* wild type or variant promoters with co-transfection of *Sp1* or *USF1*. As a result, we observed that both transcription factors significantly increased *OCT3* transcription (Fig. 3).

### Effects of the *OCT3* haplotype on the pharmacokinetics of metformin

After genotyping the *OCT3* variants, we found that 43 subjects among the 45 that participated in a previous metformin pharmacokinetics study^12^ had only H1 or H2 *OCT3* haplotypes (19 were H1/H1, 18 were H1/H2, and 6 were H2/H2). We subdivided those 43 subjects into 2 groups by H2 haplotype, which yielded significantly higher luciferase activity in the reporter assay compared to wild type; the variant group consisted of subjects homozygous or heterozygous for H2, and the control group consisted of subjects homozygous for H1. One of the remaining 2 subjects was H2/H3, and the other was H1/H5. The former was included in the variant group because this subject had haplotype H2. Additionally, because H3 consists of only the c.-29G > A variant, we can infer that this haplotype will cause higher luciferase activity, although we did not directly measure it in this study. Finally, we included the subject with the H1/H5 genotype in the control group because H5 showed a luciferase activity comparable with that of the wild type (Supplementary Fig. S2). Thus, 25 subjects were included in the variant group, and the remaining 20 subjects were assigned to the control group.

**Figure 4:**
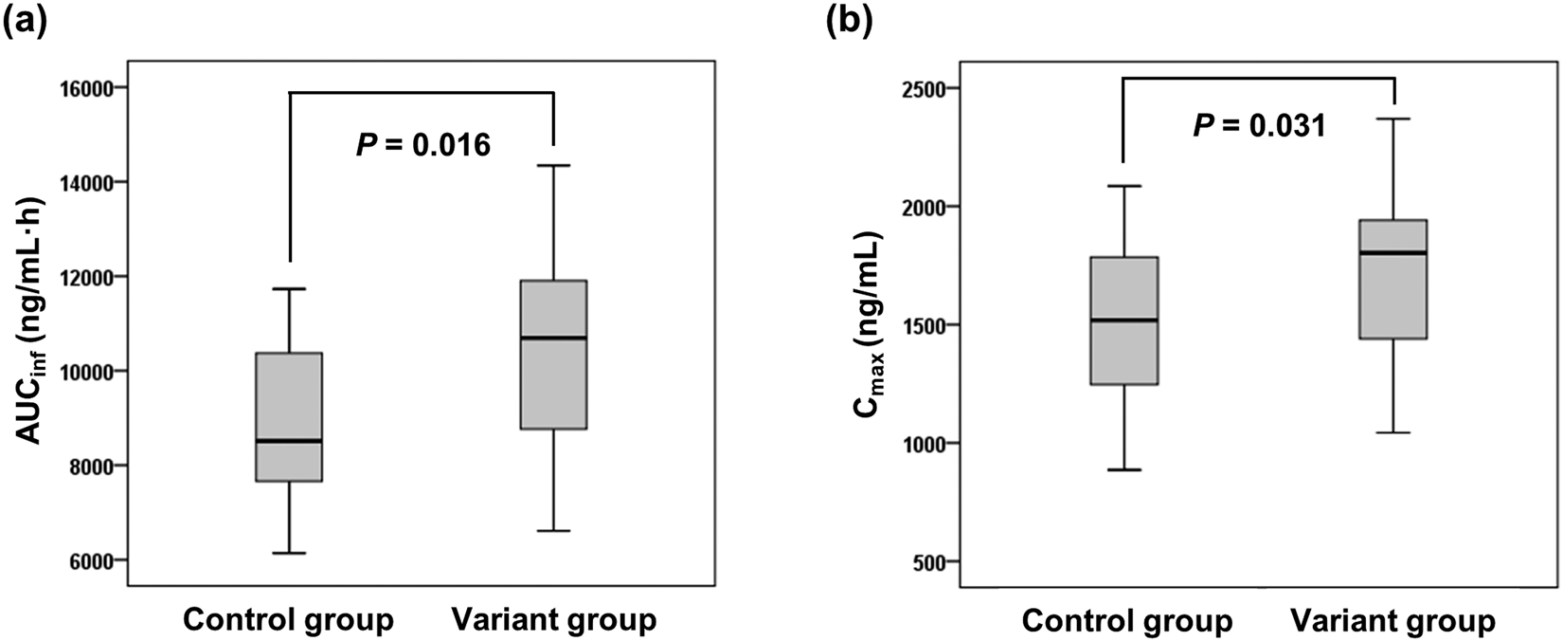
A box-and-whisker plot of pharmacokinetic parameters, AUC_inf_ (**a**) and C_max_ (**b**) after oral administration of metformin, as a function of the *OCT3* haplotype (control group, n = 20; variant group, n = 25). The horizontal lines within each box represent the median. The box edges show lower (25th) and upper (75th) quartiles. The whiskers extend from the 25th and 75th quartiles to the furthest data point within a distance of 1.5 interquartile ranges from the 25th and 75th quartiles. P*-*values were calculated by the Mann–Whitney test.

First, we compared the demographic characteristics of the subjects between the 2 groups and observed that there were no significant differences in age, sex, height, weight, and creatinine clearance under the influence of H2 (Table 3). In a previous study^12^, we reported that the functional promoter haplotypes of *MATE2K* are significantly associated with pharmacokinetics of metformin in Koreans. Therefore, we compared the frequencies of those *MATE2K* haplotypes between *OCT3* control and variant groups to exclude the effect of *MATE2K* haplotypes on the pharmacokinetics of metformin. As a result, we found that there was no significant difference in these frequencies between the two groups (*P* = 0.821, Supplementary Table S2). We also analyzed metformin pharmacokinetic data according to *MATE1* rs2252281, *MATE1* rs2289669, and *OCT2* rs316019 genotypes, which are known to affect metformin pharmacokinetics or pharmacodynamics^11,15,21^. As a result, we found that none of these variants significantly affected the pharmacokinetics of metformin in our study population (data not shown). In addition, we observed that the frequencies of these variants were not significantly different between *OCT3* control and variant groups (*P* = 1.000, 0.187, and 0.286, respectively, Supplementary Table S2). Therefore, we could exclude *OCT2*, *MATE1*, and *MAT2K* variants as confounding factors when analyzing metformin pharmacokinetic data according to *OCT3* haplotypes. Then, we compared metformin pharmacokinetics between the 2 *OCT3* groups and found that the plasma metformin concentration versus the time curve from 0 to extrapolated infinite time (AUC_inf_) was significantly higher in the variant group compared to the control group(10493.77 ± 2033.52 vs. 8950.21 ± 1684.45 ng/(mL·h); *P* = 0.016; geometric mean ratio (GMR) (90% confidence interval, CI) = 1.17 (1.06–1.29), Table 4). In addition, the subjects in the variant group showed significantly higher maximum metformin concentrations (C_max_) compared to the control group (1729.10 ± 336.94 vs. 1492.15 ± 340.70 ng/mL; *P* = 0.031; GMR (90% CI) = 1.17 (1.04–1.31), Table 4). There was no significant difference in the apparent bioavailability (F, AUC_inf_*CL_R_/Dose), elimination half-life (t_1/2_), time of maximum concentration (T_max_), metformin renal clearance (CL_R_), and secretion clearance (SrCL_R_) between the 2 groups. Figure 4 shows a box-and-whisker plot of pharmacokinetic parameters, AUC_inf_ and C_max_, after oral administration of metformin, depending on H2. In our study population, the variant group included 6 individuals homozygous for H2 and 19 individuals heterozygous for H2. Therefore, we compared the metformin pharmacokinetics among 3 groups: the control group, H2 heterozygote group, and H2 homozygote group. As a result, we observed that the subjects homozygous for H2 showed the highest AUC_inf_ and C_max_ (11240.21 ± 2399.73 vs. 10258.05 ± 1915.61 vs. 8950.21 ± 1684.45 ng/(mL·h), *P* = 0.044 and 1821.63 ± 306.49 vs. 1699.88 ± 348.60 vs. 1492.15 ± 340.70 ng/mL, *P = *0.052, respectively, Supplementary Table S3).

**Table 3:** Demographic characteristics of healthy participants according to the *OCT3* haplotype.

Parameter	Control group (n = 20)	Variant group (n = 25)	*P*
Age (year)^a^	26.90 ± 3.89	26.60 ± 4.83	0.526
Sex (male)	18	23	1.000
Height (cm)^a^	173.23 ± 6.90	171.91 ± 6.54	0.599
Weight (kg)^a^	67.51 ± 10.16	67.10 ± 8.48	0.802
CL_Cr_ (mL/min)^a^	98.14 ± 11.98	102.66 ± 13.64	0.309

^a^The data shown represent mean arithmetic values ± SD.

P-values were calculated by the Mann–Whitney test.

**Table 4:** Pharmacokinetic parameters of metformin in healthy participants according to the *OCT3* haplotype.

Parameter	Control group (n = 20)	Variant group (n = 25)	*P*	GMR (90% CI)
F	0.41 ± 0.09	0.46 ± 0.10	0.105	1.11 (1.00–1.24)
AUC_inf_ (ng/mL·h)	8950.21 ± 1684.45	10493.77 ± 2033.52	0.016	1.17 (1.06–1.29)
C_max_ (ng/mL)	1492.15 ± 340.70	1729.10 ± 336.94	0.031	1.17 (1.04–1.31)
T_max_ (h)^a^	1.50 (1–3)	1.50 (1–4)	0.191	1.21 (0.98–1.49)
t_1/2_ (h)	6.60 ± 1.98	6.75 ± 2.68	0.837	1.01 (0.86–1.19)
CL_R_ (mL/min)	581.09 ± 97.12	551.25 ± 88.84	0.385	0.95 (0.87–1.03)
SrCL_R_ (mL/min)	482.95 ± 103.15	448.59 ± 87.25	0.304	0.93 (0.84–1.03)

The data shown represent arithmetic mean values ± SD.

^a^T_max_ parameters were shown as median (range).

P-values were calculated by the Mann–Whitney test.

GMR, geometric mean ratio; CI, confidence interval.

### Effect of the *OCT3* haplotype on pharmacodynamics of metformin

We next investigated the glucose-lowering effect of metformin according to the *OCT3* haplotypes using oral glucose tolerance test (OGTT) data in healthy Koreans from our previous clinical study^12^. The glucose-lowering action of metformin was determined by the difference in pharmacodynamic parameters between before and after metformin administration. As a result, we found that there was no significant difference in the difference in the maximum glucose level (∆G_max_), the difference in the area under the serum glucose concentration versus time curve (∆G_mean_), and the difference in the area under the glucose curve spanning 0–60 minutes after glucose ingestion (∆G_mean0_–_60_) between the control and variant groups (Table 5).

**Table 5:** Glucose-lowering effect of metformin according to the *OCT3* haplotype.

Parameter	Control group (H1, n = 20)	Variant group (H2, n = 25)	*P*
∆G_max_ (mg/dL)	36.85 ± 16.78	30.60 ± 12.37	0.156
∆G_mean_ (mg/dL)	9.25 ± 9.51	5.35 ± 6.98	0.239
∆G_mean0_–_60_ (mg/dL)	16.26 ± 12.66	10.43 ± 11.78	0.283

The data shown represent arithmetic mean values ± SD.

P-values were calculated by the Mann–Whitney test.

## Discussion

This study was carried out to identify genetic variants in the *OCT3* promoter in Koreans and functionally characterize these variants by *in vitro* assays. Previously, genetic analysis for identifying *OCT3* promoter variations was conducted through direct sequencing of DNA samples from various ethnic groups. In the study, the proximal region (positions −706 to +223 relative to the translational start site) of the *OCT3* promoter was sequenced^10^. In our study, we screened a large region surrounding the promoter (positions −2,159 to +54 relative to the translational start site) of *OCT3* using DNA samples obtained from 48 healthy Koreans. In our study population, 2 haplotypes showed high frequencies, and 1 of them, H2, yielded significantly higher luciferase activity in a reporter assay as compared to that of the wild type. H2 contains 3 variants, c.-1603G > A, c.-1547T > G, and c.-29G > A. The luciferase activities of the 2 variants, c.-1603G > A and c.-1547T > G, were significantly decreased, whereas the activity of another variant, c.-29G > A, was significantly increased.

It has been shown that the transcription factor Sp1 is involved in the transcriptional effect of the variant c.-29G > A on *OCT3*^10^. Nonetheless, the mechanism through which transcription factors mediate the decreased luciferase activities of c.-1603G > A or c.-1547T > G has not been determined. Therefore, we predicted transcription factors that could bind to the promoter region of *OCT3* in the vicinity of each variant using software tools Consite and JASPAR, and confirmed these predictions by EMSAs. As a result, we found that 2 transcription factors, Sp1 and USF1, mediated the transcriptional effects of c.-1603G > A and c.-1547T > G, respectively, on *OCT3*. Sp1 is ubiquitously expressed in mammalian cells and acts as an activator or repressor during diverse processes such as cell growth, differentiation, apoptosis, angiogenesis, and immune response^25–28^. Sp1 binds to GC boxes and serves as an activator of a dopamine transporter and OCT3 transporter, as found in the present and other studies^10,29^. USF1 is also expressed ubiquitously and participates in various processes such as the stress and immune response, cell cycle, and glucide-lipid metabolism^30^. USF1 binds to the E-box regulatory elements and regulates transcriptional activities of several transporters including OCT2 and MATE1^31–33^. In this study, we observed that both transcription factors act as inducers of the *OCT3* promoter. The decreased luciferase activity of the c.-1603A variant can result from a reduction in the binding affinity of Sp1. In the case of c.-1547T > G, the variant c.-1547G showed a decreased luciferase activity, although the binding affinity of USF1 was stronger for the variant, compared to wild type. This finding indicates that other transcription factors besides USF1 are involved in the reduced transcriptional activity of c.-1547T > G.

It was demonstrated that metformin is moved through a number of transporters including OCT1–3, MATE1, and MATE2K^6^. The OCT3 transporter is expressed on the apical membrane of the intestine and mediates the uptake of organic cations^34^. Shirasaka *et al*.^24^ reported that AUC_0-∞_ and the oral bioavailability of metformin are significantly different between wild type (Oct3^+/+^) and *Oct3* knockout (Oct3^−/−^) mice; AUC_0-∞_ and bioavailability of metformin are significantly higher in wild type mice. In another study, the oral bioavailability of metformin was significantly decreased in *Oct3* knockout mice, compared to that in wild type mice, although the plasma concentrations of metformin were not significantly different between the 2 groups^35^. Therefore, in the present study, we evaluated the effect of the *OCT3* promoter haplotype on the systemic exposure and renal clearance of metformin. As a result, we observed that subjects homozygous or heterozygous for haplotype H2 showed a significant increase in AUC_inf_ and C_max_ (17.2%, and 15.9%, *P* = 0.016 and 0.031, respectively), compared to those in the control group. In particular, the subjects homozygous for H2 were found to have the highest AUC_inf_ and C_max_. In our study, the 90% CIs of the GMRs for AUC_inf_ and C_max_ were 1.06–1.29 and 1.04–1.31, respectively, and were close to, but did not fall within, the conventional bioequivalence range of 0.8–1.25^36^; the upper limit of the 90% CI of the GMRs for AUC_inf_ and C_max_ slightly exceeded 1.25. This finding indicates that the clinical effect of H2 on the pharmacokinetics of metformin is marginal. However, this increase may be of clinical significance in patients with renal impairment or other compromised conditions. Further clinical studies are necessary to determine whether this increase is clinically significant. In addition, the apparent bioavailability of metformin was higher in the variant group, compared to that of the control group, although statistical significance was not observed (0.46 ± 0.10 vs. 0.41 ± 0.09, *P* = 0.105). In particular, the apparent bioavailability in the subjects homozygous for H2 was higher than that in the subjects heterozygous for H2 (0.48 ± 0.09 vs. 0.45 ± 0.10). Therefore, the increased pharmacokinetic parameters AUC_inf_ and C_max_ in the variant group may be due to increased intestinal absorption of metformin, because of the higher expression of OCT3 and its higher transporter activity in the apical membrane of the intestine. On the other hand, the CL_R_ and SrCL_R_ of metformin were comparable between the control and variant groups. Tzvetkov *et al*.^20^ also reported no significant association between the renal clearance of metformin and 6 variants of *OCT3* in their study population. Most absorbed metformin is eliminated via renal excretion^37^, and several transporters including OCT2, MATE1, and MATE2K participate in its excretion; the uptake of metformin from the circulation to epithelial cells in renal proximal tubules is predominantly performed by OCT2^38^. Then, metformin in the cells is transported into the lumen by MATE1 and MATE2K^37,39,40^. The data in our present and previous^20^ studies imply that the renal excretion of metformin is not significantly affected by OCT3.

To exclude the effect of the other transporters, *OCT2*, *MATE1*, and *MATE2K*, on the pharmacokinetics of metformin, we compared frequencies of the SNPs in these transporters that are known to affect pharmacokinetics or pharmacodynamics of metformin and found that there was no significant difference in the frequencies of variations in the *OCT3* control and variant groups^11,15,21^. Previously it was reported that several *OCT1* variants significantly affected the pharmacokinetics or pharmacodynamics of metformin^18,19^. However, all these variants have not been identified in Asians, including Koreans^17,41^. Recently Chen *et al*.^41^ reported that 3 variations of *OCT1* identified in Asians (Chinese or Japanese populations) showed altered function in *in vitro* assays. However, we did not consider the *OCT1* genotype as a confounding factor in the analysis of metformin pharmacokinetic data in our study population, since the minor allele frequencies of these variations were very low (0.017, 0.023, and 0.008, respectively).

There are some limitations of this study. First, the sample size in the analysis of metformin pharmacokinetics depending on the *OCT3* haplotype was not sufficient for desirable statistical power since the analysis was conducted retrospectively. Nevertheless, our study is strengthened by the fact that we performed a genotype-phenotype association analysis to validate our hypothesis by the data from *in vitro* assays. To validate our data, additional association studies with greater numbers of DNA samples are necessary. Second, we could not examine the expression of OCT3 in the intestine as a function of its haplotypes. Müller *et al*.^34^ demonstrated the expression of OCT3 in the apical membrane of the human intestine by immunocytochemical analysis. However, to our knowledge, no study has evaluated OCT3 expression in the intestine depending on *OCT3* genotype. Third, there was no significant difference in pharmacodynamic parameters between the *OCT3* control and variant groups in our study population. This null effect may reflect a true lack of effect of *OCT3* haplotypes on metformin response, but it is also possible that we could not detect a genetic effect because our observations were limited to a relatively short time period in healthy volunteers. In our previous study, functional *MATE2K* promoter haplotypes also did not affect the pharmacodynamics of metformin, although there were significant differences in the metformin pharmacokinetic parameters, CL_R_ and SrCL_R_ (*P* = 0.006 and 0.007, respectively)^12^. On the other hand, in the studies by Stocker *et al*.^11^ and Choi *et al*.^13^, the differences in CL_R_ and SrCL_R_ according to *MATE2K* genotype were smaller than those we reported in our previous study^12^, but this genotype was significantly associated with the metformin response as determined by the relative change in glycated hemoglobin (HbA1_C_) in type 2 diabetes patients. Therefore, to confirm the effect of *OCT3* haplotypes on the pharmacological activity of metformin, further clinical study is necessary in patients with diabetes.

In conclusion, we found that a common *OCT3* promoter haplotype regulates transcriptional activity of this promoter and influences metformin pharmacokinetics, in particular, metformin absorption in Koreans. To our knowledge, this is the first study to assess the effect of an *OCT3* promoter haplotype on the pharmacokinetics of metformin. Further studies in various ethnic populations are necessary to confirm the contribution of this haplotype to the interindividual variability in pharmacokinetics of metformin. In addition, the investigation of metformin response according to *OCT3* promoter haplotype in patients with diabetes is necessary to determine the clinical usefulness of this haplotype.

## Methods

### Genetic analysis of the *OCT3* promoter

The study protocol was approved by the Institutional Review Board of Ewha Womans University Medical Center, Seoul, Korea. All experiments and analyses were performed in accordance with the relevant guidelines and regulations of the Institutional Review Board of the Ewha Womans University Medical Center. Genomic DNA samples were obtained from 48 healthy Koreans (after obtaining written informed consent) from the DNA bank of the Korea Pharmacogenomics Research Network at Seoul National University, Seoul, Korea. To identify genetic variations, the promoter region of *OCT3* (positions −2,159 to +54 relative to the translational start site) was amplified and directly sequenced on an ABI 3730xl DNA analyzer (Thermo Fisher Scientific, Waltham, MA, USA). Then, haplotype assembly was conducted using Haploview 4.3 software (Broad Institute, Cambridge, MA, USA). Nucleotide positions were assigned relative to the translational start site according to the *OCT3* mRNA sequence (GenBank accession number: NM_021977.3). In addition, each variation was named according to the Human Genome Variation Society (HGVS) nomenclature^42^.

### Construction of *OCT3* reporter plasmids

The reporter plasmid containing the *OCT3* wild type promoter (positions −1,642 to +15 relative to the translational start site) was amplified and inserted into the pGL4.11 [*luc2P*] vector (Promega Corporation, Madison, WI, USA). Next, the reporter plasmids containing variants of the *OCT3* promoter were generated by means of the QuikChange® II Site-Directed Mutagenesis Kit (Agilent Technologies, Santa Clara, CA, USA). Primers for the construction of reporter plasmids are listed in Supplementary Table S4. All DNA sequences of the vectors were confirmed by direct sequencing.

### Measurement of *OCT3* promoter activity

Forty-eight hours after transfection of the reporter plasmids containing either wild type or variants of *OCT3* into HCT-116 (human colon carcinoma) cells using Lipofectamine LTX and Plus reagents (Life Technologies, Carlsbad, CA, USA), luciferase activity of each vector was measured using a Dual-Luciferase® Reporter Assay System (Promega). In order to examine the effect of Sp1 or USF1 on the promoter activity of *OCT3*, *OCT3* reporter plasmids were co-transfected with different amounts (0, 25, and 50 ng) of the *Sp1*-pcDNA3.1 or *USF1*-pcDNA3.1 plasmids into HCT-116 cells. *Sp1* cDNA (GE Healthcare Dharmacon, Inc., Lafayette, CO, USA) and *USF1* cDNA (Thermo Fisher Scientific) were subcloned into the pcDNA3.1(+) vector (Life Technologies). The primers used for construction of *Sp1*-pcDNA3.1 or *USF1*-pcDNA3.1 plasmids are shown in Supplementary Table S4. The amount of a transfected plasmid was normalized to the pGL4.74 *Renilla* vector (Promega), and relative luciferase activity was calculated from a value obtained from the firefly luciferase to Renilla luciferase ratios.

### EMSA

Fifteen to 20 μg of a nuclear extract from HCT-116 cells was incubated with ^32^P-labeled (1 × 10^5^ or 2 × 10^5^ counts/min) oligonucleotides. Competition and supershift assays were performed using a 100-fold molar excess of Sp1 or USF1 consensus oligonucleotides and Sp1 (sc-420X, Santa Cruz Biotechnology, Dallas, TX, USA) or USF1 antibodies (sc-229X, Santa Cruz Biotechnology), respectively. Then, each sample was subjected to electrophoresis for 90 min at 80 V, and CP-BU film was exposed to the dried gel (Agfa, Mortsel, Belgium) at −80 °C for 16 hours. ImageJ software (National Institutes of Health, Bethesda, MD, USA) was used for the measurement of each band’s intensity. All oligonucleotides used in the EMSAs are listed in Supplementary Table S4.

### Analysis of genotypes and metformin pharmacokinetics or pharmacodynamics

Analysis of metformin pharmacokinetics or pharmacodynamics as a function of the *OCT3* haplotype was conducted retrospectively using data from our clinical trial^12^. Briefly, after written informed consent was obtained, 45 healthy participants received a 1,000 mg oral dose of metformin (Diabex Tab; Daewoong Pharmaceutical Corporation, Seoul, Korea) at 8 PM (Day 1) and a 750 mg dose of metformin at 8 AM (Day 2). To determine metformin pharmacokinetics, blood samples were collected before the second dose of metformin and after 0.5, 1, 1.5, 2, 2.5, 3, 3.5, 4, 6, 8, 10, 12, and 24 h. In addition, urine samples were collected during the following time intervals: hours 0–4, 4–8, 8–12, and 12–24 after the second metformin dose. Concentrations of metformin in plasma and urine samples were determined by a highly specific and sensitive method of liquid chromatography with tandem mass spectrometry (API 3200; Life Technologies). Pharmacokinetic parameters were calculated by noncompartmental analysis in Phoenix WinNonlin 6.1 software (Pharsight Corporation, St. Louis, MO, USA). To assess metformin response, the OGTT was conducted twice, once before (Day 1) and once after metformin administration (Day 2). In the present study, to determine *OCT3* haplotypes of each participant, we genotyped *OCT3* variants in DNA samples, while *OCT2*, *MATE1*, and *MATE2K* genotype data for each participant were obtained from our previous studies^12,33^.

### Statistical analysis

Results are presented as the arithmetic mean ± standard deviation (SD). Statistical analyses were performed using GraphPad Prism 5.0 (GraphPad Software, Inc., La Jolla, CA, USA) and SPSS v.23.0 (IBM Corporation, Armonk, NY, USA). P*-*values for the luciferase assay were calculated by one-way analysis of variance followed by Dunnett’s two-tailed test. To examine the effect of the *OCT3* haplotype on metformin pharmacokinetics, the Mann–Whitney or Kruskal–Wallis nonparametric tests were carried out. GMR and 90% CI of the GMR were calculated to estimate clinically significant differences in the pharmacokinetic parameters according to the *OCT3* haplotypes with standard bioequivalence boundaries of 80–125%^43^. In addition, we compared the frequencies of *OCT2*, *MATE1*, and *MATE2K* variations between the *OCT3* control and variant groups using the χ^2^-test. Differences were considered statistically significant at p*-*values < 0.05.

## Electronic supplementary material

Supplementary Information
