# Supplementary material for: OCT3 promoter haplotype is associated with metformin pharmacokinetics in Koreans
Source: Sci Rep. 2018 Nov 16;8:16965. doi: 10.1038/s41598-018-35322-6 (PMC6240047; doi:10.1038/s41598-018-35322-6)
Supplement: Supplementary file 1 — Supplementary Information [file 41598_2018_35322_MOESM1_ESM.pdf]

Supplementary Information For

OCT3 promoter haplotype is associated with metformin pharmacokinetics in Koreans

Eun Young Kwon[1,3], Jae-Yong Chung[2,3], Hyo Jin Park[1], Bo Min Kim[1], Minsuk Kim[1], Ji Ha Choi[1,*]

[1]Department of Pharmacology, Tissue Injury Defense Research Center, College of Medicine, Ewha Womans University, Seoul, Korea

[2]Department of Clinical Pharmacology and Therapeutics, Seoul National University College of Medicine and Bundang Hospital, Seoul, Korea

[3]EYK and JC contributed equally to this study.

[*]Corresponding author: jihachoi@ewha.ac.kr

Corresponding author

Ji Ha Choi, M.D., Ph.D.

Department of Pharmacology, Tissue Injury Defense Research Center, College of Medicine, Ewha Womans University, Seoul, Korea

1071 Anyangcheon-ro, Yangcheon-gu, Seoul, 07985, Korea

Phone: +82-2-2650-5746; Fax: +82-2-2653-8891; E-mail: jihachoi@ewha.ac.kr

Supplementary Figure S1. Linkage disequilibrium structures of *OCT3* promoter variations

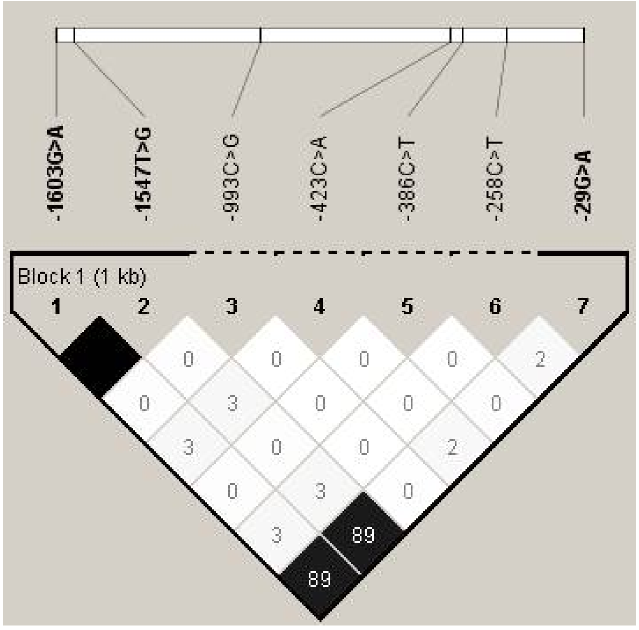

The number in the diamond indicates the r^2 value. A black diamond without a number means an r^2 value of 1.

Supplementary Figure S2. Luciferase activity of the *OCT3* promoter haplotype H5

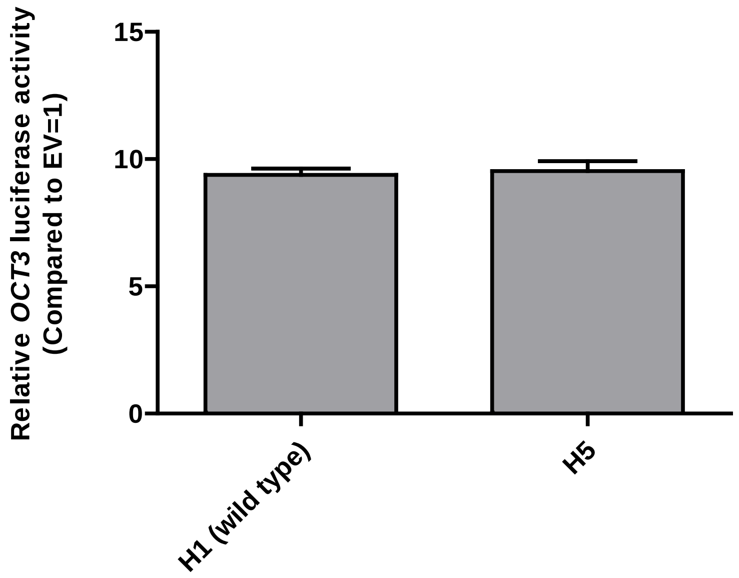

Supplementary Figure S3. Full-length images of the cropped gels presented in the main Figure 2

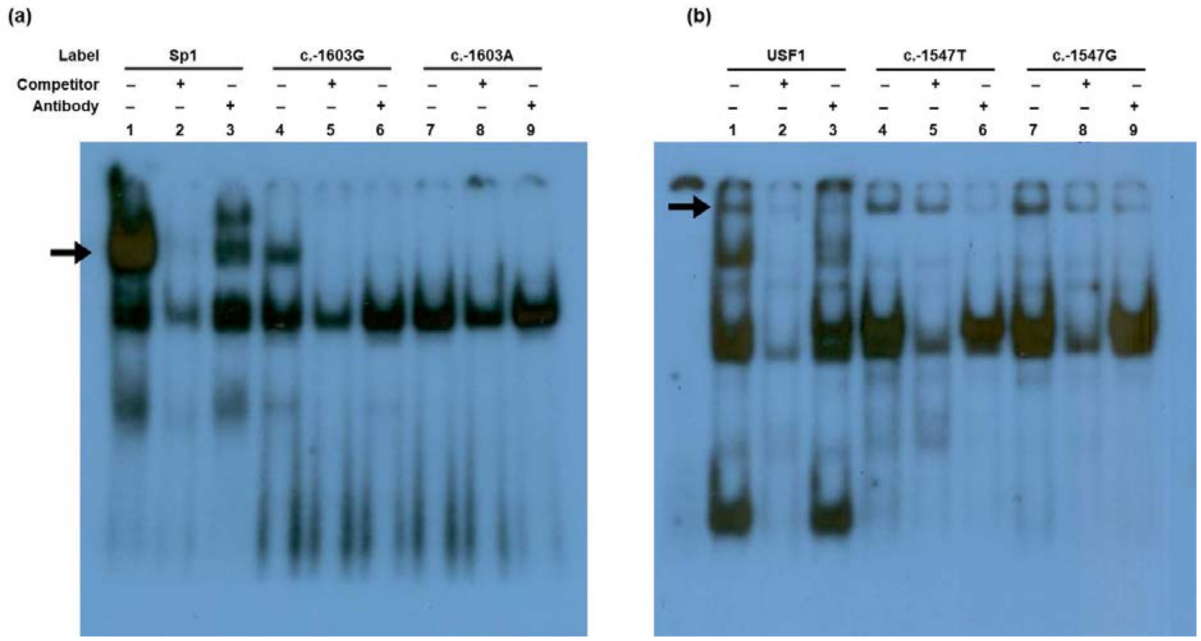

(a) Full-length image of Figure 2a shows the interaction between Sp1 and c.-1603G>A variant. Nuclear extracts were incubated with ^{32}P-labeled oligonucleotides (Sp1 consensus, lanes 1–3; c.-1603G wild type, lanes 4–6; c.-1603A variant, lanes 7–9). Competition assays and supershift assays were conducted with 100-fold molar excess of Sp1 consensus oligonucleotides (lanes 2, 5, and 8) and Sp1 antibody (lanes 3, 6, and 9), respectively. **(b)** Full-length image of Figure 2b shows the interaction between USF1 and c.-1547T>G variant. Nuclear extracts were incubated with ^{32}P-labeled oligonucleotides (USF1 consensus, lanes 1–3; c.-1547T wild type, lanes 4–6; c.-1547G variant, lanes 7–9]. Competition and supershift assays were conducted with 100-fold molar excess of USF1 consensus oligonucleotides (lanes 2, 5, and 8) and USF1 antibody (lanes 3, 6, and 9), respectively. The arrows indicate the position of the DNA–protein complexes.

Supplementary Table S1. Frequencies of *OCT3* genetic variations in promoter region from ASW/CEU/CHB population data

rs Number	Variation	Minor allele	Frequency		
			ASW	CEU	CHB
rs520685	c.-1603G>A	A	0.139	0.237	0.272
rs520829	c.-1547T>G	G	0.516	0.475	0.272
rs555754	c.-29G>A	A	0.492	0.480	0.291

Data was obtained from 1000 Genomes Project (phase 3).

ASW (American of African Ancestry in SW USA), CEU (Utah Residents with Northern and Western European Ancestry), CHB (Han Chinese in Beijing, China).

Supplementary Table S2. Frequencies of *MATE2K, MATE1, OCT2* functional promoter haplotypes or variants between *OCT3* control and variant groups

Genotype	*OCT3*		*P*
	control group (n=20)	variant group (n=25)	
MATE2K control group	15	18	0.821
MATE2K variant group[1]	5	7	
MATE1 rs2252281 wild type	15	19	1.000
MATE1 rs2252281 variant[2]	5	6	
MATE1 rs2289669 wild type	3	8	0.187
MATE1 rs2289669 variant[2]	17	17	
OCT2 rs316019 wild type	8	14	0.286
OCT2 rs316019 variant[2]	12	11	

[1]The variant group included individuals who are homozygous for *MATE2K* haplotypes 1 or 2, which showed increased promoter activities in the previous *in vitro* assay.

[2]The variant group included individuals who are heterozygous or homozygous for a minor allele.

P-values were calculated by the χ^2-test.

Supplementary Table S3. Pharmacokinetic parameters of metformin according to the *OCT3* haplotype

Parameter	Control group (n=20)	Variant group (n=25)		*P*
		H2 heterozygote (n=19)	H2 homozygote (n=6)	
F	0.41 ± 0.09	0.45 ± 0.10	0.48 ± 0.09	0.208
AUC_{inf} (ng/mL·h)	8950.21 ± 1684.45	10258.05 ± 1915.61	11240.21 ± 2399.73	0.044
C_{max} (ng/mL)	1492.15 ± 340.70	1699.88 ± 348.60	1821.63 ± 306.49	0.052
T_{max}[1] (h)	1.50 (1-3)	1.50 (1-3)	1.75 (1-4)	0.327
$t_{1/2}$ (h)	6.60 ± 1.98	7.14 ± 2.82	5.52 ± 1.85	0.289
CL_R (mL/min)	581.09 ± 97.12	554.89 ± 85.78	539.74 ± 105.75	0.671
$SrCL_R$ (mL/min)	482.95 ± 103.15	453.08 ± 84.28	434.36 ± 103.21	0.559

The data shown represent arithmetic mean values ± SD.
[1]T_{max} parameters were shown as median (range).
P-values were calculated by the Kruskal–Wallis test.

Supplementary Table S4. Oligonucleotides used in the construction of *OCT3* reporter, *Sp1*, and *USF1* plasmids or in EMSA

Primes for *OCT3* promoter cloning[1] (-1,642 to +15; 1,657bp)	
Sense (XhoI site)	5'-GTG C**CT CGA G**GC CTC TCT GAG CAG CCT TTT-3'
Antisense (HindIII site)	5'-CGC C**AA GCT T**GT CGA AGG AGG GCA TGG TG-3'
Primes for *Sp1* cloning[1]	
Sense (HindIII site)	5'- **AAG CTT** GTT CGC TTG CCT CGT CAG C -3'
Antisense (XhoI site)	5'- **CTC GAG** TCC CGG GGT TAA GGG GTA TG -3'
Primes for *USF1* cloning[1]	
Sense (NheI site)	5'- **GCT AGC** GGA TGT GCC CCC TCA CAG AG -3'
Antisense (XhoI site)	5'- **CTC GAG** GCC CAA AGC CCC TGA ATC C -3'
Primers for *OCT3* mutagenesis PCR[2]	
c.-1603G>A	5'-CCT TCT GGG TAA GGG **A**CA GGT TCT TCT GTG G-3'
c.-1547T>G	5'-GCA ATC AAC AAG GTA GAA CAG **G**TG ATT TCT TTA TGG CCA GTT T-3'
c.-29G>A	5'-CCG AGG CGC G**A**G CTG CGG GCG-3'
Oligonucleotides in EMSA	
c.-1603G (wild type)[2]	5'-CTG GGT AAG GG**G** CAG GTT CTT C-3'
c.-1603A (variant)[2]	5'-CTG GGT AAG GG**A** CAG GTT CTT C-3'
Sp1 consensus[3]	5'-ATT CGA TC**G GGG CGG GGC** GAG C-3'
c.-1547T (wild type)[2]	5'-AAG GTA GAA CAG **T**TG ATT TCT TTA T-3'
c.-1547G (variant)[2]	5'-AAG GTA GAA CAG **G**TG ATT TCT TTA T-3'
USF1 consensus[3]	5'-GTG T**AG GCC ACG TGA CCG** GGT GTA AGC TTC-3'

[1]The restriction endonuclease sites were marked by bold-faced letters with underlines.

[2]The SNP sites were marked by bold-faced letters with underlines.

[3]The consensus sequences of transcription factors were marked by bold-faced letters with underlines.
